# Characterization of RNA in exosomes secreted by human breast cancer cell lines using next-generation sequencing

**DOI:** 10.7717/peerj.201

**Published:** 2013-11-05

**Authors:** Piroon Jenjaroenpun, Yuliya Kremenska, Vrundha M. Nair, Maksym Kremenskoy, Baby Joseph, Igor V. Kurochkin

**Affiliations:** 1 Department of Genome and Gene Expression Data Analysis, Bioinformatics Institute, Singapore; 2 Interdisciplinary Research Centre, Malankara Catholic College, Mariagiri, Kaliakkavilai, Tamil Nadu, India

**Keywords:** Exosomes, Microvesicles, Next generation sequencing, Breast cancer, Biomarkers

## Abstract

Exosomes are nanosized (30–100 nm) membrane vesicles secreted by most cell types. Exosomes have been found to contain various RNA species including miRNA, mRNA and long non-protein coding RNAs. A number of cancer cells produce elevated levels of exosomes. Because exosomes have been isolated from most body fluids they may provide a source for non-invasive cancer diagnostics. Transcriptome profiling that uses deep-sequencing technologies (RNA-Seq) offers enormous amount of data that can be used for biomarkers discovery, however, in case of exosomes this approach was applied only for the analysis of small RNAs. In this study, we utilized RNA-Seq technology to analyze RNAs present in microvesicles secreted by human breast cancer cell lines.

Exosomes were isolated from the media conditioned by two human breast cancer cell lines, MDA-MB-231 and MDA-MB-436. Exosomal RNA was profiled using the Ion Torrent semiconductor chip-based technology. Exosomes were found to contain various classes of RNA with the major class represented by fragmented ribosomal RNA (rRNA), in particular 28S and 18S rRNA subunits. Analysis of exosomal RNA content revealed that it reflects RNA content of the donor cells. Although exosomes produced by the two cancer cell lines shared most of the RNA species, there was a number of non-coding transcripts unique to MDA-MB-231 and MDA-MB-436 cells. This suggests that RNA analysis might distinguish exosomes produced by low metastatic breast cancer cell line (MDA-MB-436) from that produced by highly metastatic breast cancer cell line (MDA-MB-231). The analysis of gene ontologies (GOs) associated with the most abundant transcripts present in exosomes revealed significant enrichment in genes encoding proteins involved in translation and rRNA and ncRNA processing. These GO terms indicate most expressed genes for both, cellular and exosomal RNA.

For the first time, using RNA-seq, we examined the transcriptomes of exosomes secreted by human breast cancer cells. We found that most abundant exosomal RNA species are the fragments of 28S and 18S rRNA subunits. This limits the number of reads from other RNAs. To increase the number of detectable transcripts and improve the accuracy of their expression level the protocols allowing depletion of fragmented rRNA should be utilized in the future RNA-seq analyses on exosomes. Present data revealed that exosomal transcripts are representative of their cells of origin and thus could form basis for detection of tumor specific markers.

## Introduction

Exosomes are nanosized membrane vesicles secreted by multiple cell types (Raposo & Stoorvogel, 2013). The term “exosomes” was introduced in 1987 by Johnstone et al. (1987) to describe the “trash” vesicles released by differentiating reticulocytes to dispose unwanted membrane proteins which are known to diminish during maturation of reticulocytes to erythrocytes. Later exosomes have been implicated in more complex functions. Raposo et al. (1996) have demonstrated that exosomes derived from B lymphocytes activate T lymphocytes, suggesting a role for exosomes in antigen presentation *in vivo*. In early studies, secreted microvesicles were named based on their cellular origins, e.g., archaeosomes, argosomes, dexosomes, epididymosomes, prostasomes, oncosomes, etc. (Raposo & Stoorvogel, 2013). More lately it became apparent that vesicles released by the same cell type are heterogeneous and can be classified into at least three classes based on their mode of biogenesis: (1) exosomes (30–130 nm in diameter), which originate from multivesicular endosome (MVE); (2) microvesicles (100–1000 nm in diameter), which are shed from the plasma membrane; (3) apoptotic bodies (1–4 µm in diameter), which are released from fragmented apoptotic cells during late stages of cell death (Raposo & Stoorvogel, 2013). Various purification procedures including sequential centrifugation protocols have been proposed to separate these vesicles for further analysis. Biochemical and proteomic analyses showed that exosomes contain specific protein set reflecting their intracellular site of formation. Exosomes from different cell types contain endosome-associated proteins, e.g., tetraspanins (CD9, CD63, CD81, CD82), annexins, Rab GTPases and flotillin. Exosomes are also enriched in proteins involved in MVE formation (Tsg101 and Alix), chaperones (Hsc73 and Hsc90) and cytoskeletal proteins (Raimondo et al., 2011). Furthermore, exosomes contain proteins that are specific to the cells from which they are derived (Choi et al., 2012; Sandvig & Llorente, 2012).

The research in the exosome field has exploded rapidly after the discovery that these microvesicles transport a large number of mRNA and miRNA (Valadi et al., 2007) and that exosomal mRNAs could be translated into proteins by recipient cells (Ratajczak et al., 2006; Valadi et al., 2007) and exosomal miRNAs are able to modulate gene expression in recipient cells (Mittelbrunn et al., 2011). Interestingly, certain mRNAs and miRNAs were identified as highly enriched in exosomes compared to that of the host cells indicating the existence of selective sorting mechanism controlling incorporation of RNA into exosomes (Skog et al., 2008; Valadi et al., 2007). Previously, we demonstrated that exosomal RNAs share specific sequence motifs that may potentially function as *cis*-acting elements for targeting to exosomes (Batagov, Kuznetsov & Kurochkin, 2011).

Given the fact that exosomes carry complex biological information consisting of proteins, lipids and RNAs, it is not surprising to find that they have been implicated in a variety of physiological and pathological conditions. Skokos et al. (2001) reported, for example, that mast cells communicate with other cells of the immune system via exosomes promoting mitogenic activity in B and T lymphocytes. A number of studies have demonstrated the role of exosomes in the development of the nervous system, synaptic activity, neuronal regeneration, neuron-glia communication and protection against injury (Lai & Breakefield, 2012). Furthermore, exosomes can be involved in the pathogenesis of cancer and degenerative diseases. The fact that tumor cells release a large amount of exosomes was initially demonstrated in ovarian cancer patients (Taylor, Homesley & Doellgast, 1983). Exosomes were shown to be secreted by various tumor cells including those derived from breast (King, Michael & Gleadle, 2012), colorectum (Silva et al., 2012), brain (Graner et al., 2009), ovarian (Escrevente et al., 2011), prostate (Mitchell et al., 2009; Nilsson et al., 2009), lung (Rabinowits et al., 2009), and bladder (Welton et al., 2010) cancer. A significantly higher amount of exosomes was found in plasma from lung cancer patients compared to that of control individuals (Rabinowits et al., 2009). In colorectal cancer patients, the amount of plasma circulating exosomes was constitutively higher than in normal healthy individuals showing the direct correlation between exosomes quantity and malignancy (Silva et al., 2012).

Manipulating tumor cells to decrease the release of exosomes followed by their injection into immunocompetent mice led to a significantly slower tumor growth compared to that of unperturbed cells (Bobrie et al., 2012). It has been shown that exosomes which are released from tumor cells are able to transfer a variety of molecules, including cancer-specific, to other cells (Al-Nedawi, Meehan & Rak, 2009; Muralidharan-Chari et al., 2009) to manipulate their environment, making it more favorable for tumor growth and invasion. Glioblastoma-derived exosomes were found to be enriched in angiogenic proteins that allowed them to stimulate angiogenesis in endothelial cells (Skog et al., 2008). Melanoma exosomes were shown to be instrumental in melanoma cell dissemination via alterations in the angiogenic microenvironment. Hood et al. demonstrated that metastatic factors responsible for the recruitment of melanoma cells to sentinel lymph nodes are upregulated by melanoma exosomes themselves (Muralidharan-Chari et al., 2009). Exosomes shed by human MDA-MB-231 breast carcinoma cells and U87 glioma cells were capable of conferring the transformed characteristics of cancer cells onto normal fibroblasts and epithelial cells in part due to transferring tissue transglutaminase and fibronectin (Hood, San & Wickline, 2011).

Because of their small size exosomes have an ability to penetrate intercellular contacts to reach distant parts of the body with the help of the blood stream and other body fluids. Exosomes have been purified from human plasma, serum, bronchoalveolar fluid, urine, tumoral effusions, epididymal fluid, amniotic fluid and breast milk (Raposo & Stoorvogel, 2013). Since exosomes possess characteristic protein and RNA signatures of their host cells, analysis of exosomes in various body fluids can be potentially utilized for non-invasive diagnostics of cancer and other disorders. For example, aggressive human gliomas often express a truncated and oncogenic form of the epidermal growth factor receptor, known as EGFRvIII. The tumour-specific EGFRvIII was detected in serum microvesicles from glioblastoma patients (Skog et al., 2008). High stability of exosomal RNA (Skog et al., 2008; Valadi et al., 2007) and ease of RNA detection by highly sensitive PCR makes detection of exosomal RNA an attractive approach for the discovery of biomarkers. Indeed, mRNA variants and miRNAs characteristic of gliomas could be detected in serum microvesicles of glioblastoma patients (Skog et al., 2008). Expression profiles of serum microvesicle mRNA by microarrays correctly separated individuals with glioblastoma from normal controls (Noerholm et al., 2012). Of all RNA species, secreted miRNAs were most frequently utilized toward discovery of body fluid-based biomarkers perhaps because miRNA expression profiles are more informative than mRNA expression profiles in a number of diseases (Grady & Tewari, 2010). In one study, analysis of plasma- and serum-derived microvesicles revealed 12 miRNAs differently expressed in prostate cancer patients compared to that of healthy controls and 11 miRNAs upregulated in patients with metastases compared to that of patients without metastases (Bryant et al., 2012). Interestingly, exosomes released by breast cancer cells can be separated into different classes depending on their miRNAs content (Palma et al., 2012). Cells undergoing malignant transformation produced exosomes containing selective miRNAs, whose release is increased by malignant transformation, in contrast to cells that are not affected by malignancy, whose exosomes are packed with neutral miRNAs (Palma et al., 2012). The changes in exosomal miRNA cargo could provide a signature of the presence of malignant cells in the body.

The majority of reported to date exosomal miRNA and mRNA profiles have been generated using microarray approaches that suffer from several limitations. Microarrays are biased for investigation of already discovered transcripts. In addition, there is potential for cross-hybridization of RNAs that are highly related in sequence. Recently developed next generation RNA sequencing technology (RNA-Seq) allows detection of all RNA subtypes as well as of unannotated transcripts and has a high sensitivity toward identification of low-abundance RNAs. In case of exosomes, this approach was applied only for the analysis of small (20–70 nt) RNAs (Bellingham, Coleman & Hill, 2012; Nolte-’t Hoen et al., 2012).

In this study, we utilized the RNA-Seq approach to characterize the transcriptomes of exosomes secreted by two metastatic human breast cancer cell lines. We describe optimized computational workflow to analyze data generated by the Ion Torrent semiconductor chip-based technology. We have identified and profiled RNA species present in exosomes and host cells and discuss the utility of exosomal RNA as potential breast cancer-specific biomarkers.

## Materials and Methods

### Cell culture

Human breast cancer cell lines MDA-MB-436 (ATCC^®^ HTB-130™) and MDA-MB-231 (ATCC^®^ HTB-26™) were maintained at 37°C in 5% CO2 and cultured in DMEM/F12 supplemented with 10% FBS. 48 h prior to exosome collection, cells were washed 3 times with PBS and the medium was changed to serum-free CCM5 medium (Thermo Scientific).

### Exosome extraction

Exosomes were isolated and purified from the media of MDA-MB-436 and MDA-MB-231 cell cultures using sequential centrifugation protocol. Briefly, media was collected and cellular debris was removed by centrifugation at 3,000×g for 10 min. The supernatant was centrifuged at 17,000×g for 30 min at 4°C. The supernatant was collected and centrifuged at 100,000×g for 2 h to pellet exosomes. Exosomes pellets were then washed in filtered PBS and re-centrifuged at 100,000×g, the supernatant was removed and the final exosomal pellet was re-suspended in 100 µl PBS.

### Transmission electron microscopy

A 50 µl aliquot of exosomes was absorbed onto formvar carbon coated nickel grid for 1 h. The grid was positioned with the coating side facing the drop containing exosomes. Then the grids were washed by sequentially positioning them on top of the droplets of 0.1 M sodium cacodylate, pH 7.6 and then fixed in 2% paraformaldehyde and 2.5% glutaraldehyde in 0.1 M sodium cacodylate, pH 7.6 for 10 min. Then grids were washed again with 0.1 M sodium cacodylate, pH 7.6 and contrasted with 2% uranyl acetate in 0.1 M sodium cacodylate, pH 7.6 for 15 min. After washing, the grids were incubated on top of the drop of 0.13% methyl cellulose and negatively stained with 0.4% uranyl acetate for 10 min, air dried for 5 min and examined with a JEM-2200FS transmission electron microscope operated at 100 kV.

### Nanoparticle tracking analysis

Supernatants containing vesicles were analyzed using a Nano-Sight LM10 instrument equipped with a 405 nm laser (NanoSight, Amesbury, UK) at 25°C. Particle movement was tracked by NTA software (version 2.2, NanoSight) with low refractive index corresponding to cell-derived vesicles. Each track was then analyzed to get the mean, mode, and median vesicle size together with the vesicle concentration (in millions) for each size.

### RNA isolation and analysis

Total RNA from exosomes (MDA-MB-436 and 231) and cultured cells (MDA-MB-231) were isolated using the TRIzol reagent (Invitrogen). RNA quality and concentration were assessed with the Agilent 2100 Bioanalyzer (Thermo Scientific). Cellular RNA was analyzed using RNA 6000 Nano Kit (Agilent) and exosomal RNA was analyzed using RNA 6000 Pico kit (Agilent).

### RNA-seq with ion torrent personalized genome machine (PGM)

Two hundreds ng of exosomal RNA and 3 µg of total cell RNA was used as the starting input for RNA-Seq library preparation. Sequencing was performed by AITbiotech company (Singapore). Briefly, total cell RNA was treated with RiboMinus Eukaryote kit (Life Technologies) to remove rRNA. Then, exosomal and rRNA-depleted cellular RNAs were fragmented using RNaseIII. Whole transcriptome library was constructed using the Ion Total-RNA Seq Kit v2 (Life Technologies). Bar-coded libraries were quantified with qRT–PCR. Each library template was clonally amplified on Ion Sphere Particles (Life Technologies) using Ion One Touch 200 Template Kit v2 (Life Technologies). Preparations containing bar-coded libraries were loaded into 318 Chips and sequenced on the PGM (Life Technologies).

### cDNA synthesis and quantitative real-time PCR (RT-PCR)

Two-step RT-PCR was performed using the QuantiTect Reverse Transcription Kit (QIAGEN GmbH, Hilden, Germany) according to manufacturer’s protocol. RT-PCR was performed in a Rotor-Gene (Qiagen) using a SYBR Green PCR Master Mix. Primer sequences are provided in Table S3. All reactions with template and without template (negative controls) were run in duplicate and averaged. GAPDH was used as internal control for mRNA. C*t* value was detected for each gene meaning the cycle number at which the amount of amplified gene of interest reaches a fixed threshold. Relative quantification (fold change) was determined for the host cells and exosomal genes expression and normalized to an endogenous control GAPDH relative to a calibrator as 2^−ΔΔC*t*^ (where ΔC = (C*t* of gene of interest) – (C*t* of endogenous control gene (GAPDH) and ΔΔC*t* = (ΔC*t* of samples for gene of interest) – (ΔC*t* of calibrator for the gene of interest). Melting curves of each amplified products were analyzed to ensure uniform amplification of the PCR products.

### Bioinformatics analysis

#### Raw reads filtering

Raw reads generated by sequencing were subjected to several quality checks. The low quality reads were removed by read trimming and read filtering. Read trimming included removal of adapter sequences, removal of the 3′ ends with low quality scores and trimming based on High-Residual Ionogram Values. Filtering of entire reads included removal of short reads, adapter dimers, reads lacking sequencing key, reads with off-scale signal and polyclonal reads. Subsequent analysis was performed with high quality reads which passed through the described above filtering steps.

#### Reads mapping

Bowtie 2 version 2.1.0 was used to align all high-quality reads to rRNA sequences including 28S (NR_003287.2), 18S (NR_003286.2), 5S (NR_023379.1), and 5.8S (NR_003285.2) rRNA. Reads mapped to rRNA sequences were filtered out while the rest of the reads were mapped to the human genome. The high-quality reads were mapped to hg19 build of the human genome from University of California Santa Cruz (UCSC) genome browser database (Meyer et al., 2013) using TopHat version 2.0.6 with the aligner Bowtie 2.0.5 (Kim et al., 2013; Langmead & Salzberg, 2012) with their default parameters in end-to-end mode (-b2-sensitive) and defining splice-junctions based on known splice-junctions (-G). To classify the reads into known and unknown genes, the BAM file generated by Tophat2 was intersected to known gene (RefGene and GENCODE built V14 from UCSC database) using BEDtools (Quinlan & Hall, 2010) and was used to count the number of reads by SAMtools (Li et al., 2009).

#### Post-processing of the aligned reads

The mapped reads were further manipulated by removing the reads that mapped to multiple locations. In particular, the short aligned reads with the length of <20 nucleotides were eliminated to avoid the alignment errors such as mapping to multiple genomic locations. Further filtering included the removal of the low quality reads which fall below the mapping quality score of 10 (-q 10) using SAMtools. For the coverage search, the BAM file generated by Tophat2 was converted to BED format with option (-split) using BEDtools. The BED file was converted again to BAM format using BEDtools. We then developed python script (using pysam as part of the scripts) to calculate the number of reads and read coverage in exons and protein-coding sequence (CDS) regions consecutively.

#### RNA abundance calculation

RNA abundance was estimated with the help of Partek Genomics Suite software (Partek Inc., St. Lous, MO) using Reference Sequence Gene (RefSeq Gene) and GENCODE annotation built version 14 of those not overlapped with RefSeq Gene from UCSC genome browser. The Expectation-Maximization (E/M) Algorithm (Xing et al., 2006) was used to estimate the most likely relative expression levels of each gene isoform. Partek’s algorithm was used to quantify the gene isoform expression level as reads per kilo base per million mapped reads (RPKM).

#### Functional analysis of genes

Database for Annotation, Visualization, and Integrated Discovery (DAVID) (version 6.7) was used to identify gene functional annotation terms that are significantly enriched in particular gene lists with the whole Human genes as the background (Huang, Sherman & Lempicki, 2009). A list of gene symbols was generated for each dataset and was used as input into DAVID. DAVID calculates a modified Fishers Exact *p*-value to demonstrate Gene ontology (GO) and KEGG molecular pathway enrichment, where *p*-values less than 0.05 after Benjamini multiple test correction are considered to be strongly enriched in the annotation category. We also used a count threshold of 5 and the default value of 0.1 for the EASE (enrichment) score settings. We used more specific GO term categories provided by DAVID, called GO FAT, to minimize the redundancy of general GO terms in the analysis to increase the specificity of the terms.

## Results

### Characterization of exosomes released by breast cancer cells

**Figure 1:**
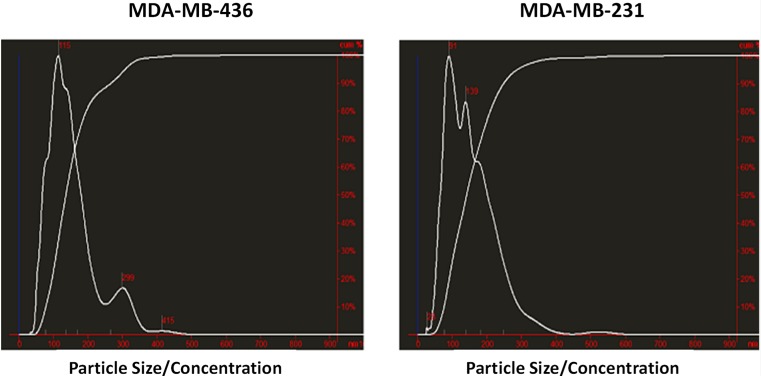
Analysis of exosomes produced by breast cancer cell lines, MDA-MB-436 and MDA-MB-231, with Nanosight LM10-HS instrument.

**Figure 2:**
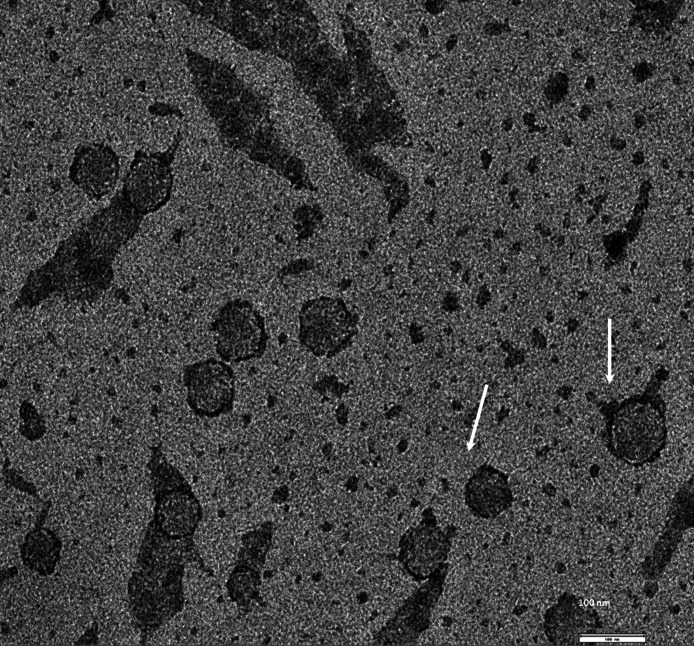
TEM image of the exosomes produces by MDA-MB-436 cell line. Electron microscopy allowed visualizing membrane-bound nanovesicles sized ∼100 nm. White arrowheads pointing to the exosomes. Scale bar = 100 nm.

Exosomes were isolated from two breast cancer cell lines, MDA-MB-436 and MDA-MB-231 using classical ultracentrifugation protocol. The size distribution and amount of exosomes were analyzed using NanoSight LM10 nanoparticle tracking analysis (NTA). NTA showed that MDA-MB-231 cells released 4 × 10^6^ vesicles per cm^2^ of growth area per 48 h that were predominantly 115 nm in size. MDA-MB-436 cells released 1.04 × 10^7^ vesicles per cm^2^ of growth area per 48 h that were predominantly 91 nm in size (Fig. 1). The size of exosomes released from both cell lines ranged from ∼70 nm to ∼300 nm. An examination of the purified vesicles using transmission electron microscopy revealed that they had the size (∼50–100 nm) and morphology (Fig. 2) typical of that of exosomes.

### Characterization of exosomal RNA

**Figure 3:**
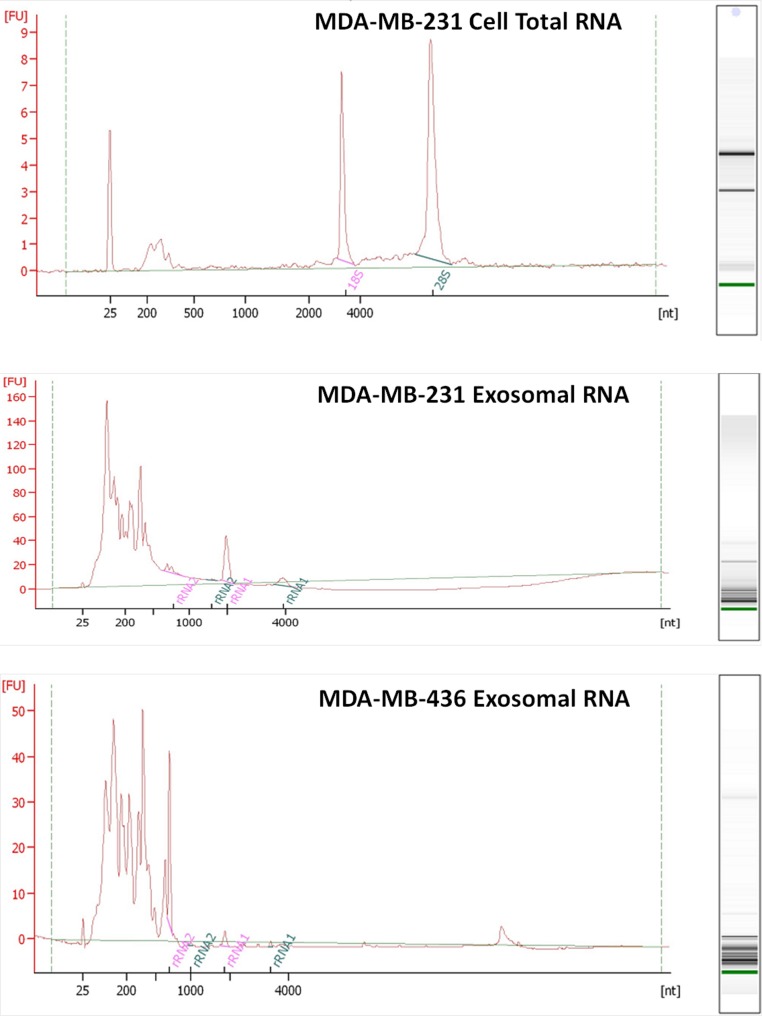
Analysis of RNA from cells and exosomes by Bioanalyzer. Exosomal and total cell RNA was analyzed with PicoChip and NanoChip, respectively.

RNA was isolated from exosomes released by both breast cancer cell lines. Total RNA was also extracted from MDA-MB-231 cell line as a control host cell line that produced exosomes. Bioanalyzer data revealed that exosomes contain a broad range of RNA sizes (30–500 nt) and have very small amount of intact rRNA (5.2% in MDA-MB-231 exosomes and 5.6% in MDA-MB-436 exosomes) (Fig. 3) consistent with previous reports on exosomal RNA (Rabinowits et al., 2009; Valadi et al., 2007).

### Exosomal RNA deep sequencing using Ion Torrent technology

We utilized the Ion Torrent sequencing technology to profile exosomal RNA produced by MDA-MB-436 and MDA-MB-231 cell lines, as well as RNA obtained from host cell line MDA-MB-231. Based on RNA quality assessment using Bioanalyzer profiling (Fig. 3) we performed rRNA depletion for cellular RNA but not for exosomal RNA.

### Identification of appropriate alignment tool for efficient capture of splice-junction reads produced by the Ion Torrent technology

**Figure 4:**
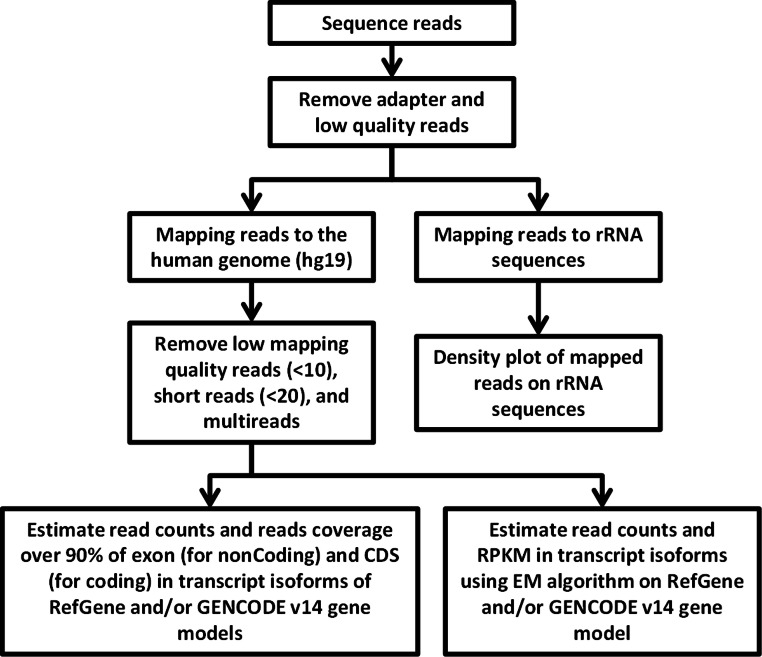
Flowchart of RNA-seq data analysis. The raw reads are exposed to pre-alignment quality checks including the removal of adaptor sequences and low quality reads. The high quality reads are then mapped to rRNA sequences using Bowtie2 version 2.1.0. The non-mapped rRNA reads were mapped to human genome hg19 build of the human genome using TopHat version 2.0.6 with the aligner Bowtie 2.0.5. After mapping, low mapping quality reads less than 10; short reads with length less than 20 base pairs and multi- reads were removed. Estimation of read counts and read coverage on mapped reads where over 90% of exon (non-coding) and CDs (for coding) in transcript isoforms of RefGene and/ or GENCODE v14 gene models. The EM algorithm along with GENCODE v14 annotations was used to estimate the read count and reads per kilo base per million mapped reads (RPKM) on mapped transcripts.

RNA-Seq computational analysis workflow is presented in Fig. 4. The single-end RNA reads generated from sequencing of the exosomal and host cell libraries were trimmed to remove adapter sequences and then filtered. After filtering out low-quality reads, the high-quality reads had a varying length ranging from 6 bp to >300 bp. These preceded high-quality reads were considered for further analysis. The total amount of reads for RNA from MDA-MB-231 cells was about 4.8 M. RNA-Seq resulted in ∼3.5 M and ∼3.2 M reads for RNA from MDA-MB-436 and MDA-MB-231 cells derived exosomes, respectively. At first, the reads were aligned to rRNA sequences including 28S, 18S, 5S, and 5.8S rRNA (see Methods). 24% of the reads in cellular and more than 80% of the reads of both exosome samples were mapped to rRNA sequences (Fig. S2). The rest of the reads were mapped to the human genome (UCSC; hg19 built) with the help of TMAP aligner program in accordance with the Ion Torrent recommended pipeline (Technologies) (data not shown). However, the use of TMAP resulted in misalignment of the splice-junction reads or reads containing multiple exons to the genome (Fig. S1). Therefore, we used alternative aligner Tophat 2.0.6 along with the Bowtie 2.0.5 for the read mapping.The mapped results are shown in Fig. S2. In total, this alignment produced more than 4.3 M (∼90%) reads of MDA-MB-231 cellular RNA that could be mapped to rRNA sequences and the human genome. For the MDA-MB-231 and MDA-MB-436 cell-derived exosomes, these alignments resulted in ∼2.8 M (∼90%) and ∼3.2 M (∼91%) of mapped reads, respectively (Fig. S2). More than 80% of mapped reads from exosomal samples were found to be mapped to rRNA sequences. We further investigated the reads of both exosomal RNA samples, which mapped to rRNA sequences. These reads were counted and plotted as read density over each rRNA sequence (shown in Fig. S3A). The mapped reads covered entire length of all rRNA sequences. The major fractions of mapped reads were 28S and 18S rRNA (Fig. S3B).

Reads mapped to multiple locations in the genome have been eliminated to obtain uniquely mapped reads. Subsequent downstream analysis was performed with the high quality reads which had the read length greater than 20 bps and mapping quality score of above 10 (see Methods). As a result, about 81, 76, and 70% out of all mapped reads were considered as high quality mapped reads for MDA-MB-231 and MDA-MB-436 exosomal RNA, and MDA-MB- 231 cellular RNA, respectively.

**Figure 5:**
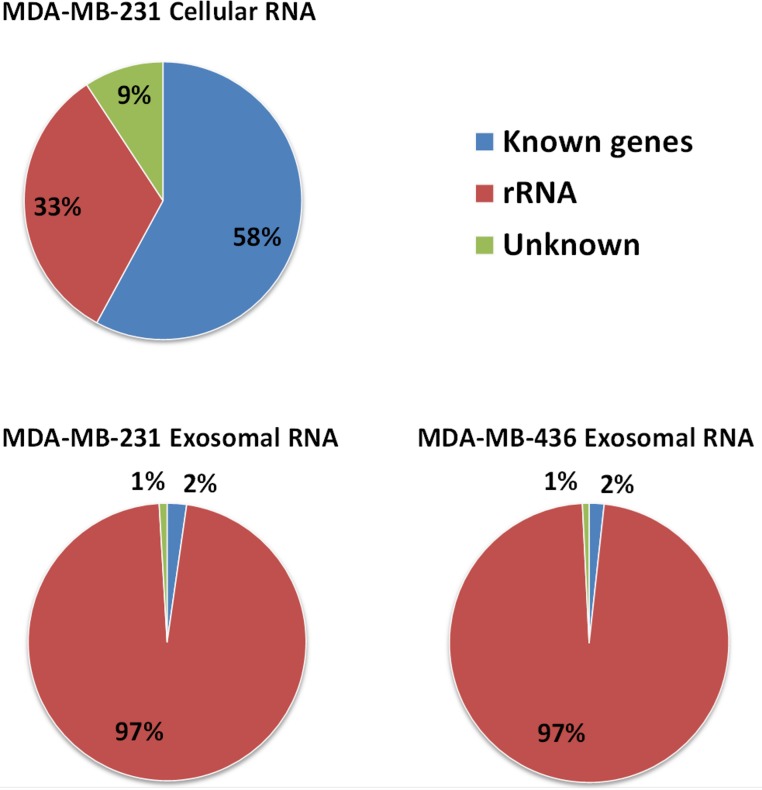
Distribution of uniquely mapped RNA-seq reads among transcriptome. Reads which overlapped with annotated gene models (RefSeq and/or GENCODE) are termed as “known genes”. Reads that placed outside of annotated gene models are termed as “unknown”. Reads which are mapped to rRNA sequences including 5S, 5.8S, 18S, and 28S rRNA are named as “rRNA”.

Approximately 97% (∼2 M reads) of reads from both exosomal RNA samples were mapped to rRNA sequences (Fig. 5) and ∼2% of the reads were mapped to known genes (RefSeq and/or GENCODE gene models). At the same time, for the MDA-MB-231 cellular RNA, for which rRNA depletion step was performed, the majority of the reads (∼58%) was mapped to known genes (Fig. 5). To increase the number of reads for other RNAs we attempted to deplete rRNA with the RiboMinus™Eukaryote Kit recommended by Ion Total RNA-Seq Kit protocol. As expected, this protocol efficiently pulled down rRNA from cellular RNA (>60%) but had little effect on removal of rRNA from exosomal RNA (Fig. S4). The failure to deplete exosomal fragmented rRNA can be explained by the design of the RiboMinus™Probe. It consists of 2 probes each for 5S, 5.8S, 18S and 28S RNA. Because the probe size is 22–25 nucleotides, many fragments of rRNA are not targeted.

### Content of cellular and exosomal transcriptomes

**Figure 6:**
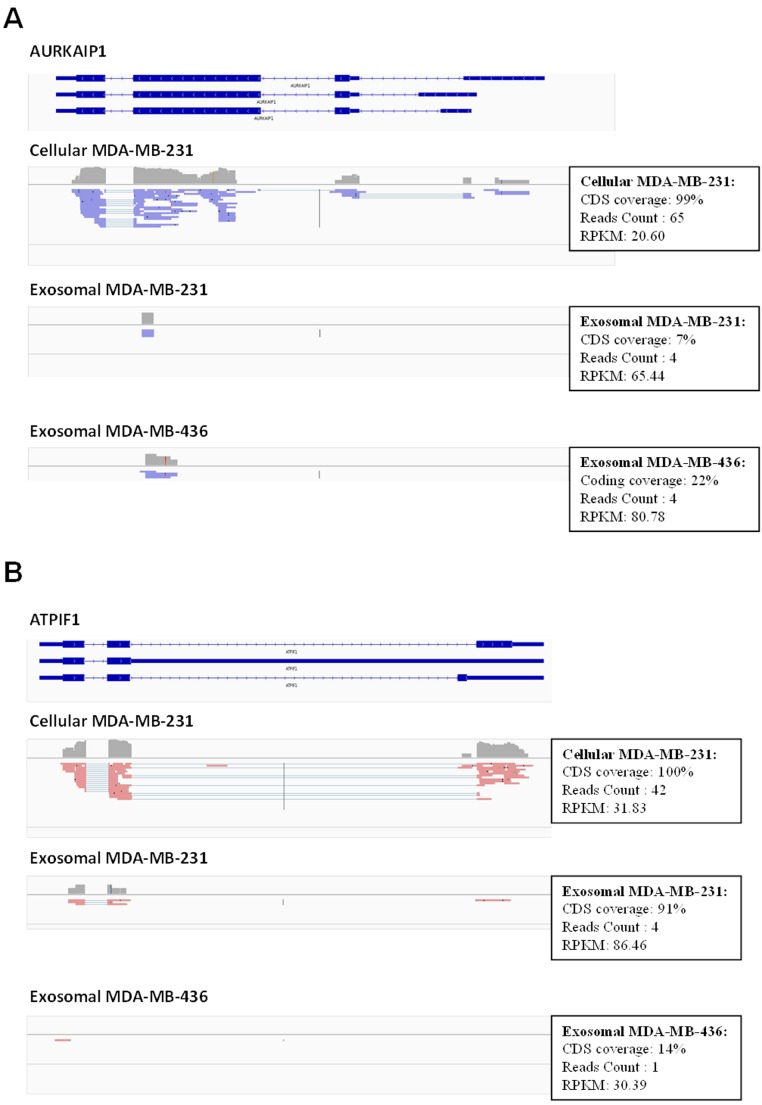
Example of low coverage transcript but very high RPKM in AURKAIP1 and ATPIF1 genes. (A) AURKAIP1 gene from chromosome position chr1:1,309,009–1,310,847 is shown using Integrative Genomic Viewer. Among the three variants, the maximum value of protein-coding sequence (CDS) coverage, read count and RPKM is shown in the right panel of read mapping. Both the exosomes shows very low coverage (7–22%) with read counts of 4, whereas the RPKM value is 65.44 and 80.78 RPKM for exosomes of MDA-MB-231 and MDA-MB-436, respectively. (B) ATPIF1 gene from chromosome position chr1:28,562,494–28,564,655 is visualized. The MDA-MB-231 exosomes exhibit high CDS coverage (91%) with an exon count of 4.

In total, 16,086 transcripts (11,657 genes) were detected with a normalized RPKM value of greater than 1.0 at least in one sample. We used Integrative Genomics Viewer (IGV) version 2.1.21 (Thorvaldsdottir, Robinson & Mesirov, 2013) to visualize mapped reads and to check the coverage across human transcriptome. We observed frequently misannotated transcripts in exosome samples with high RPKM value in those cases when only small parts of the transcripts were covered by the reads (Fig. 6). This was the effect of low depth sequencing caused by the presence of a large amount of reads representing rRNA. Therefore, we implemented more stringent criteria to obtain full-length expressed genes by filtering genes based on the RNA reads coverage. Reads which cover over 90% of protein-coding sequence (CDS) of protein-coding genes and over 90% of exons in non-coding sequence of non-coding genes were considered for further analysis. To identify protein-coding genes in exosomal samples, the mapped reads of exosomal RNA were pooled with estimated CDS coverage of >90% for both exosomal reads. The pooled mapped reads were used to calculate CDS in each exosomal sample as >50% of coverage.

**Figure 7:**
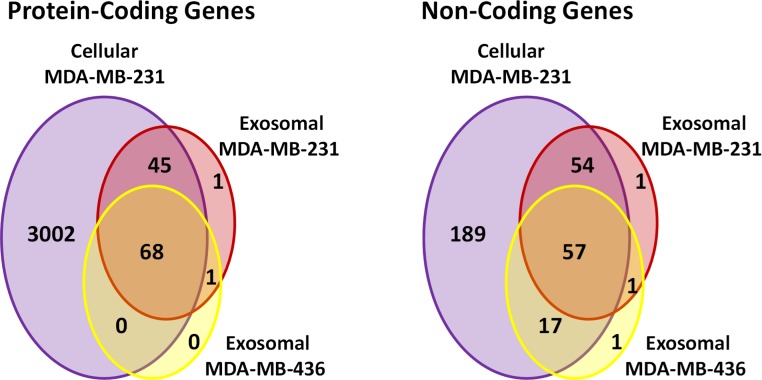
Venn diagram presents overlap among protein-coding and non-coding gene symbols in exosomes and cells. Almost all the genes in both exosomal RNA are the subset of cellular genes.

Using these criteria, we obtained lower number of annotated transcripts (6,187 transcripts or 3,437 genes) compared to that when RPKM values were considered (Fig. S5 and Table S1). As a result, some transcripts showed high coverage with CDSs criteria (for example, ATPIF1) in exosomal RNA from MDA-MB-231 cells, even when the number of reads was small (less than 5) (Fig. 6B). Such genes were also taken into consideration in our analysis. In total, 5821 (3115 genes) and 187 (115 genes) protein-coding transcripts were detected based on the RNA reads coverage in cellular and exosomal samples, respectively. For non-coding genes, 360 (317 genes) and 131 (131 genes) transcripts were detected based on the RNA reads coverage for cellular and exosomal samples, respectively. Analysis of these transcripts revealed that they represented 90.8% of protein-coding genes and 9.2% of non-coding genes for host cell sample; while exosomal RNA samples represented 50.4% and 49.6% of protein-coding and 47.6% and 52.4% of non-coding genes in MDA-MB-231 and MDA-MB-436 cells derived exosomes, respectively (Table 1). We found that 98.3% of protein-coding and 97.7% of non-coding exosomal transcripts were present in host cells (Fig. 7).

**Table 1:** Amount of genes in cellular and exosomal RNA based on 90% coverage over protein-coding sequence of genes and exons of non-coding genes. Note the large proportion of non-coding transcripts in exosomal RNA.

Transcript type	MDA-MB-231 cellular genes (%)	MDA-MB-231 exosomal genes (%)	MDA-MB-436 exosomal genes (%)
Protein-coding	90.8	50.4	47.6
Non-coding	9.2	49.6	52.4

### Exosomes are enriched in mRNAs functioning in protein translation and rRNA processing

**Figure 8:**
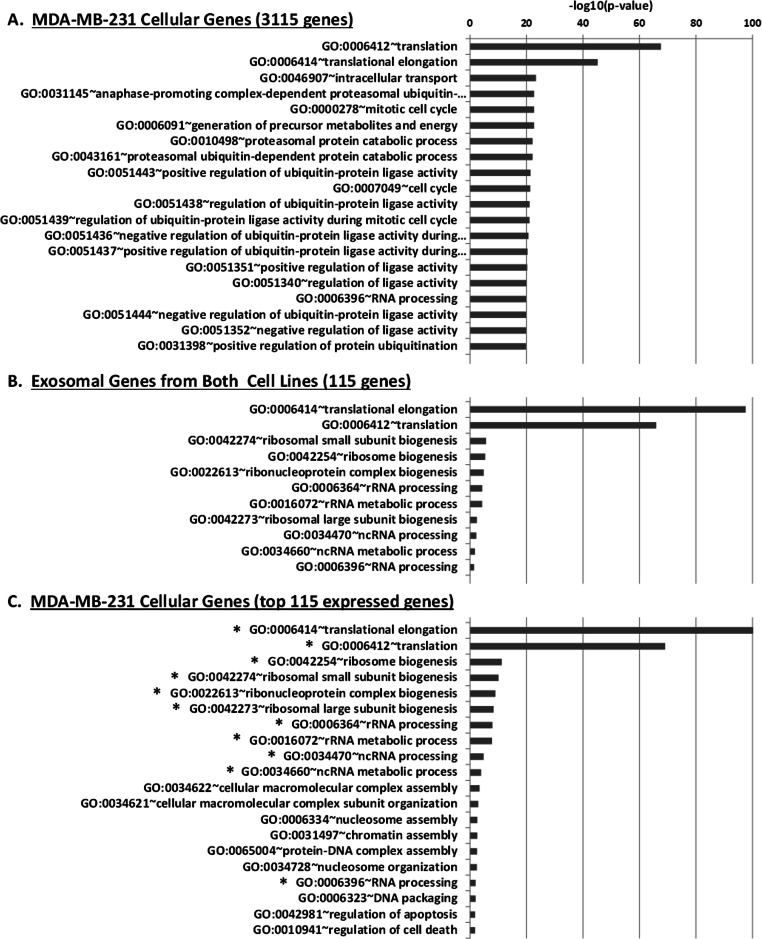
Gene Ontology (GO) enrichment analysis of genes detected in cellular and exosomal RNA from breast cancer cell lines. The significant GO terms was defined as described in Materials and Methods. (A) Top 20 significant GO terms found in MDA-MB-231 cellular genes (3115 genes). (B) Significant GO terms found in exosomal genes from both cell-lines (MDA-MB-231 and MDA-MB-436). (C) Top 20 significant GO terms found in the most expressed 115 genes from MDA-MB-231 cellular genes. The asterisks (*) indicate GO terms that present in exosomal genes.

**Figure 9:**
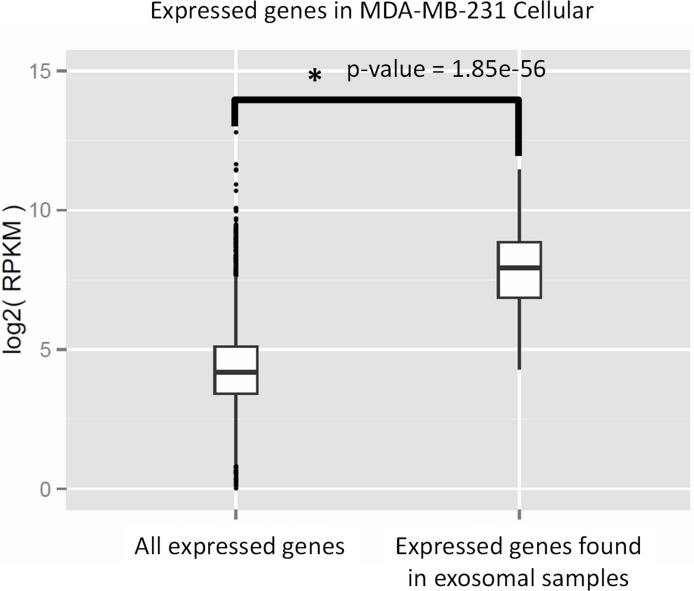
Expressed genes in exosomes found to be highly expressed in the host cells. The box plot indicates expression level of all genes in cellular samples as compared to that of genes which were found to be express in exosomes. Wilcoxon rank sum test represents significant difference in expression level of the two sets.

We performed Gene Ontology (GO) enrichment analysis using the DAVID bioinformatics resource, which employs a Fisher’s Exact Test with Benjamini–Hochberg correction. A total of 377 enriched GO categories were derived using a *P*-value cut-off of *p* < 0.05 for 3115 host MDA-MB-231 cellular genes: 286 Biological Process (BP) categories and 91 Molecular Function (MF) categories (Table S2). In total, 18 GO categories including 11 BP and 7 MF were derived from 115 exosomal genes from both cell lines. Figure 8 shows top 20 BP categories of the host cellular genes which include translation process, cell cycle, RNA processing, etc. (Fig. 8A and Table S2B). At the same time exosomal genes revealed biological processes in translation, ribosome biogenesis, rRNA and ncRNA processing GO categories (Fig. 8B and Table S2C). Since the major fraction of exosomal samples were rRNA species, significantly lower number of mRNA could be detected in exosomal samples. We hypothesized that the genes detected from exosomal samples should be highly expressed in the cells. To test the hypothesis, we performed GO enrichment analysis for 115 top expressed genes from MDA-MB-231 cellular sample. The top 10 GO terms (Fig. 8C and Table S2D) of these top expressed genes are the same as in exosomal fraction (Fig. 8B and Table S2C). We further created box plot of 115 exosomal genes in MDA-MB-231 cellular sample using expression values (RPKM) (Fig. 9). These data clearly showed that exosomes are enriched in genes which are highly expressed in the host cells.

Non-coding transcripts could be classified into 13 categories (see Table 2). Both exosomal and cellular samples contained small nucleolar RNA (snoRNA) as major species. The second most abundant class of non-coding transcripts according to GENCODE annotation was “non-coding RNA” in cellular sample and small nuclear RNA (snRNA) in exosomal samples. Overall, the top five RNA categories represented about 90% of all noncoding genes in both exosomal and cellular RNA.

**Table 2:** Amount of non-coding gene symbol in cellular and exosomal RNA based on 90% coverage over exons of non-coding transcripts. In exosomes, the top 5 non-coding gene types including small nucleolar RNA, small nuclear RNA, Mt_tRNA, microRNA, and non-coding RNA represents about 90% of non-coding genes in both exosome samples.

Gene type	MDA-MB-231 cellular (gene symbols)	MDA-MB-231 exosomal (gene symbols)	MDA-MB-436 exosomal (gene symbols)
small nucleolar RNA	214	83	51
small nuclear RNA	23	11	10
Mt_tRNA	13	7	4
microRNA	34	6	2
non-coding RNA	42	1	0
guide RNA	20	0	1
vault RNA	3	0	3
rRNA	1	1	2
RNase MRP RNA	1	1	1
RNase P RNA	1	1	1
Mt_rRNA	1	1	0
lincRNA	1	0	0
telomerase RNA	1	0	0

### Validation analysis of RNA-seq data by qRT-PCR

**Figure 10:**
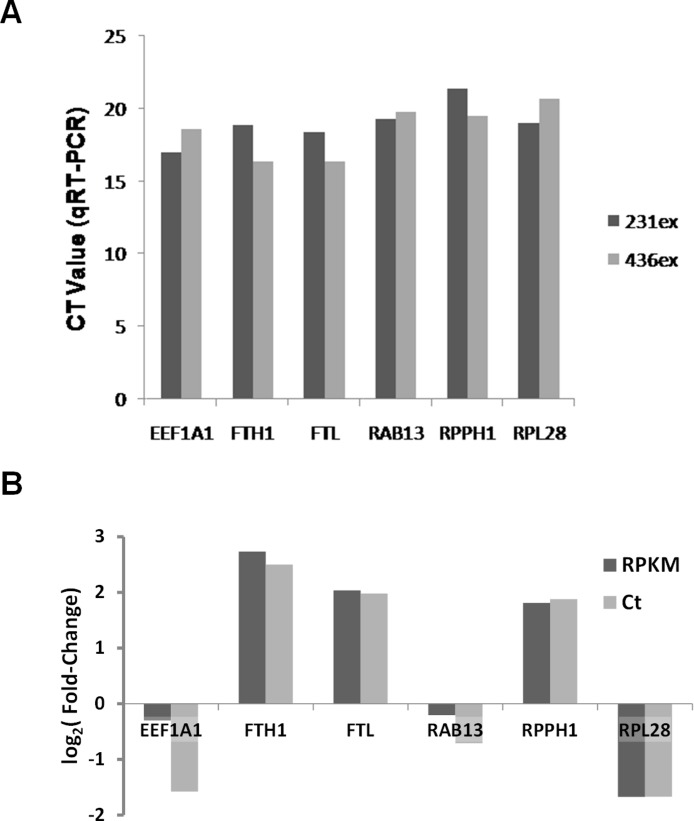
Validation of RNA-seq data by qRT-PCR. (A) Ct values for six mRNA transcripts which were detected in exosomal samples by RNA-seq are shown. (B) Comparison of different expression values (RPKM; MDA-MB-436/RPKM; MDA-MB-231) detected by RNA-Seq (dark-grey columns) and qRT-PCR (light-grey columns) for six differently expressed genes.

Based on RNA-Seq data we evaluated presence and enrichment of several mRNA transcripts in exosomal RNA - RAB13, RPPH1, EEF1A1, FTH1, FTL and RPL28. qRT-PCR analysis showed presence of all selected transcripts in exosomal samples (Fig. 10A). Figure 10B demonstrates that the fold-change of qRT-PCR results are consistent with the fold-change of RNA-seq data.

## Discussion

Until recently, the changes in gene expression during various biological processes have been analyzed using microarray approaches that focus largely on the behavior of protein-coding transcripts. Because microarrays are based on hybridization, they have high background owing to cross-hybridization, they have a limited dynamic range of detection and they rely upon known structures of genes. Development of RNA-Seq technology permitted comprehensive analysis of whole transcriptomes with the single nucleotide resolution allowing quantification of most RNA molecules expressed in the cell or tissue (Mortazavi et al., 2008). In this study, we used the Ion Torrent platform to interrogate transcriptomes of exosomes released from two metastatic breast cancer cell lines. At the time of conducting our analysis this technology produced relatively low number of reads, yet we selected it as it provided the longest reads than any other sequencing platform. This feature of the Ion Torrent technology was essential as we dealt with RNA isolated from exosomes whose nature and composition are still not well established. RNA-seq data analysis is complicated by the intricacy of dealing with large datasets, reads quality control, alignment procedure etc. Different workflows and several algorithms have been proposed to map reads to the reference genome and to perform data analysis (Chen, Wang & Shi, 2011; Mortazavi et al., 2008). Comparison of expression levels across different samples and experiments is often difficult and requires complicated normalization methods and these are still under active development. The situation is even more complex in case of exosomal transcriptomes that differ significantly from cellular transcriptomes.To address this issue, we developed in this study customized bioinformatics workflow and demonstrated its utility for analysis of exosomal RNA. Because the Ion Torrent platform produces reads with different length the dedicated algorithm for their alignment to the genome called TMAP was recommended. We found out, however, that this tool does not allow satisfactory mapping of reads that contain splice-junctions or span introns. Therefore, we choose alternative aligning tool TopHat2 (with Bowtie2) which could handle reads of varying length and identify splice-junctions based on known splice-junctions as well as allowed the discovery of new splice-junctions (Kim et al., 2013; Langmead & Salzberg, 2012).

We observed a large proportion of reads mapped to rRNA regions in exosomal samples. This was surprising given the fact that intact 18S and 28S rRNA peaks were almost undetectable in exosomal RNA (Fig. 3). This observation suggested that the majority of exosomal rRNA is fragmented. Exosomal rRNA fragments could be mapped over entire length of rRNA (Fig. S3). Fragmented 28S and 18S rRNA were major rRNA species present in exosomes. The reads mapped to 28S and 18S rRNA were distributed almost equally in exosomal and cellular RNA samples. What is the possible reason for generation of exosomal rRNA fragments? RNases present in cell culture conditioned medium are unlikely to contribute to rRNA fragmentation since exosomal membranes provide protection against RNase attack. Indeed, treatment of the exosomal preparations with RNase A did not lead to significant difference between treated and control samples in RNA size distribution (data not shown). In the study of Skog et al. (2008) RNase treatment of the glioblastoma exosomes led to a very insignificant (less than 7%) decrease in RNA suggesting that exosomal RNA is inaccessible for RNase from outside the vesicles. A possibility exists that the rRNA fragments are generated after secretion by RNases originated from the host cells and incorporated into exosome vesicles. Alternatively, rRNA fragments could be generated inside cells prior to their release to exosomes. Another class of RNA, tRNA is represented in exosomes mainly by its fragments (Nolte-’t Hoen et al., 2012). The most abundant tRNA hits in exosomal RNA are all located at the 5′ end of mature tRNAs (Nolte-’t Hoen et al., 2012).

Regardless the biogenesis of rRNA fragments, it is advisable to perform rRNA depletion step even in the absence of visible rRNA peaks on RNA profiles. This procedure would allow obtaining much higher sequencing depth for other RNA species. Our attempt to deplete fragmented rRNA with the popular RiboMinus™Eukaryote Kit failed because of the design of the probes. Because the probes size is short, many fragments of rRNA are not targeted. The use of larger number of longer probes is expected to produce a more efficient way of pulling-down fragmented rRNA. This technical aspect of working with exosomal RNA samples should be certainly considered in the future studies.

As a result of large rRNA presence in exosomal samples we observed only 2% of mapped reads to known transcripts using RefSeq and GENECODE gene models. Moreover, with RPKM value >1 we observed a large amount of misannotation due to poor coverage of the reads over transcripts. Therefore, we suggested another approach, namely filtering genes based on reads coverage over protein coding sequence for mRNA or exons for non-coding RNA. This procedure allowed us to achieve more than 90% coverage for protein-coding and non-coding regions which we considered as highly reliable for functional classification. This approach was helpful to reveal highly expressed genes in exosomes which could be potentially used as noninvasive breast cancer markers.

We report that exosomes are carrying mRNAs that are highly expressed in the host breast cancer cells (Fig. 9). Thus exosomal transcriptomes are representative of their cells of origin and should provide a platform for detection of tumor specific markers. GO analysis revealed that main biological and molecular functions of both cellular and exosomal transcripts are enriched in proteins involved in ribosome biogenesis, translational elongation, ribosomal subunit assembly and rRNA processing. What could be the significance of these functions in exosomal transcriptome? Exosome-associated mRNAs were shown to be translated into proteins by recipient cells (Ratajczak et al., 2006; Valadi et al., 2007). We hypothesize that upon arrival to the recipient cells exosomal mRNAs are translated into proteins supporting ribosomal functions to ensure efficient translation of other exosomal mRNAs within a cellular compartment where exosome content is released. Valadi et al. (2007) also described presence of mRNAs for many ribosomal proteins in exosomes secreted by mouse mast cell line. Interestingly, Graner et al. (2009) demonstrated the presence of elongation and translation factors in exosomes derived from brain tumor.

In conclusion, here we demonstrated for the first time that fragmented rRNA is a major species of exosomal RNA. Proposed here custom bioinformatics workflow allowed us to reliably detect other, non-ribosomal RNAs under conditions of limited read numbers. Classification and quantification of the RNA-Seq data revealed that exosomal transcripts are representative of their cells of origin and thus could form basis for detection of tumor specific markers. This information can also be used for getting insights in the molecular underpinnings of biological effects produced by these microvesicles. Finding that exosomes bear mRNAs encoding the necessary components to build-on-site ribosomes provides a valuable insight into biological function of these vesicles.

## Supplemental Information

10.7717/peerj.201/supp-1 Figure S1 Comparison of mapping quality between the alignment tools TopHat2.0.6 and TMAP 0.3.7 FTL gene (chromosome position chr19:49,468,467-49,470,296) is selected as an example of alignment comparison. TMAP alignment resulted in poor reads mapping and absence of junctions over exon-exon region. As the same time, TopHat identifies the exon-exon splice junctions and connects the exons through a linker.

10.7717/peerj.201/supp-2 Figure S2 Distribution of all mapped and unmapped RNA-seq reads among genomic compartments rRNA defined as 5S, 5.8S, 18S, and 28S rRNA sequences. Reads which overlapped with annotated gene models (RefSeq and/or GENCODE) are termed as “known genes”. Reads that placed outside of annotated gene models are termed as “unkown”.

10.7717/peerj.201/supp-3 Figure S3 Fragments of rRNA in exosomes represent full-length of rRNA sequence (A) RNA read density plot represents RNA fragments which fully covers of 5S, 5.8S, 18S, and 28S rRNA sequences from exosomal RNA. (B) 18S and 28S rRNA were major fractions of rRNA species.

10.7717/peerj.201/supp-4 Figure S4 Analysis of rRNA depletion from MDA-MB-231 cellular and exosomal RNA using RiboMinus™ Eukaryote Kit for RNASeq RNA was detected with PicoChip using Bioanalyzer. The depletion procedure has been performed according to the manufacturer’s protocol. Control samples (red) were treated exactly as experimental samples (blue) except they did not contain RiboMinus™ Probe.

10.7717/peerj.201/supp-5 Figure S5 Venn diagram of genes generated by RPKM and read coverage approaches The detection criteria is that gene has more than 1 RPKM in at least one sample, while another approach is that gene has more than 90% coverage over protein-coding or non-coding sequence.

10.7717/peerj.201/supp-6 Table S1 Two approaches of gene transcripts selection using RPKM or read coverage (A) The Partek genomic suite output showing transcripts with RPKM >1 in at least one sample. The read counts from the transcript isoforms were estimated using EM algorithm from Partek genomic suite on RefGene and/or GENCODE v14 gene models. (B) Estimation of read coverage (in percentage) and read count of transcript. The transcripts with >90% coverage for protein-coding sequence and exonic sequence (in case of non-coding transcript) of transcript isoforms on RefGene and/or GENCODE v14 gene models are shown.

10.7717/peerj.201/supp-7 Table S2 Gene Ontology (GO) enrichment analysis using the DAVID bioinformatics resource of Genes found in cellular and exosomal samples (A) Gene lists for GO enrichment analysis. (B) GO enrichment of cellular genes. (C) GO enrichment of exosomal genes. (D) GO enrichment of top 115 expressed cellular genes.

10.7717/peerj.201/supp-8 Table S3 List of primers used for qRT-PCR

## Additional Information and Declarations

### Data Deposition

The following information was supplied regarding the deposition of related data: http://web.bii.a-star.edu.sg/systems/piroon_data/exosome/.
