# Supplementary figures and images for: Characterization of RNA in exosomes secreted by human breast cancer cell lines using next-generation sequencing
Source: PeerJ. 2013 Nov 5;1:e201. doi: 10.7717/peerj.201 (PMC3828613; doi:10.7717/peerj.201)

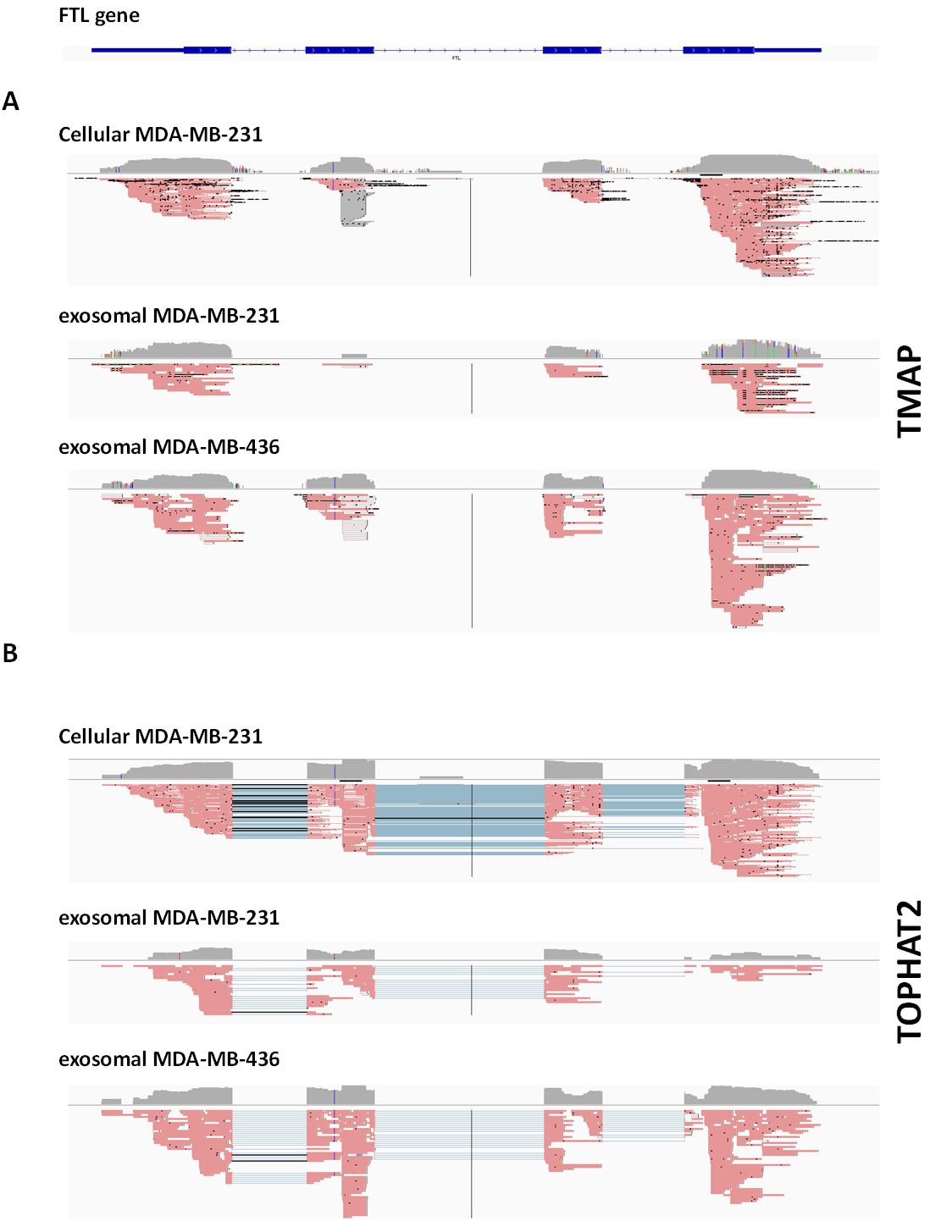

Supplement: Figure S1 — FTL gene (chromosome position chr19:49,468,467-49,470,296) is selected as an example of alignment comparison. TMAP alignment resulted in poor reads mapping and absence of junctions over exon-exon region. As the same time, TopHat identifies the exon-exon splice junctions and connects the exons through a linker. [file peerj-01-201-s001.png]

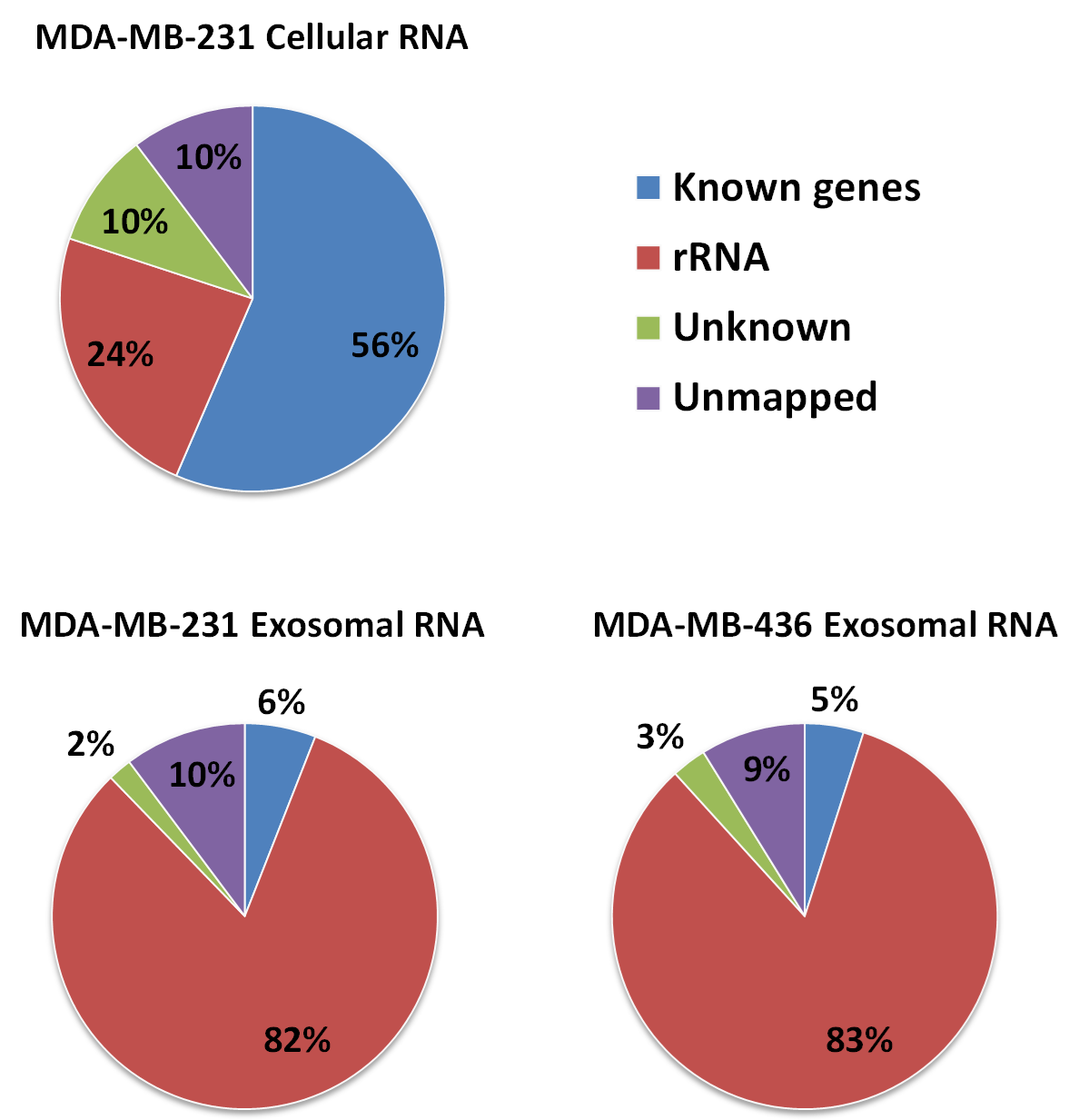

Supplement: Figure S2 — rRNA defined as 5S, 5.8S, 18S, and 28S rRNA sequences. Reads which overlapped with annotated gene models (RefSeq and/or GENCODE) are termed as “known genes”. Reads that placed outside of annotated gene models are termed as “unkown”. [file peerj-01-201-s002.png]

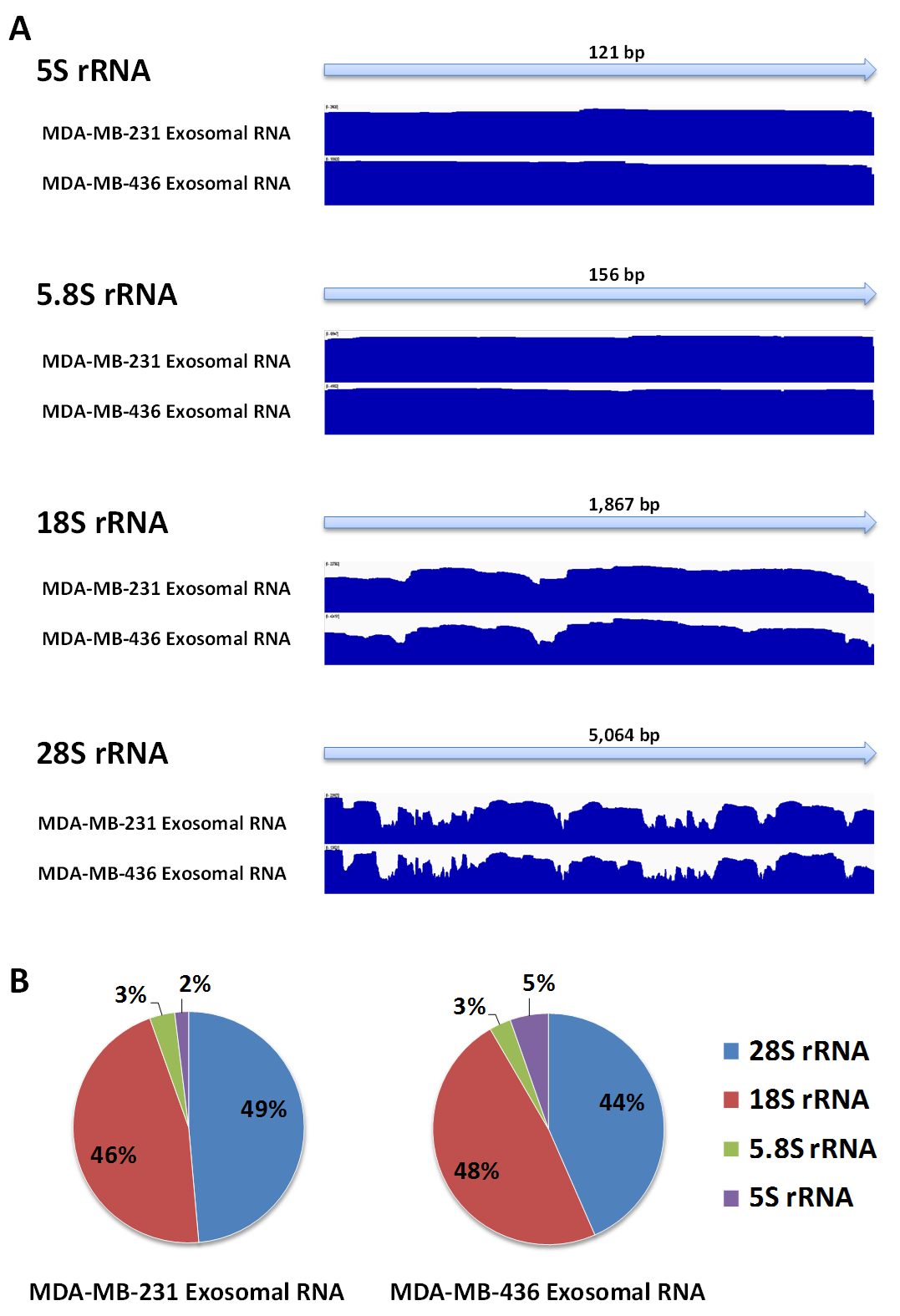

Supplement: Figure S3 — (A) RNA read density plot represents RNA fragments which fully covers of 5S, 5.8S, 18S, and 28S rRNA sequences from exosomal RNA. (B) 18S and 28S rRNA were major fractions of rRNA species. [file peerj-01-201-s003.png]

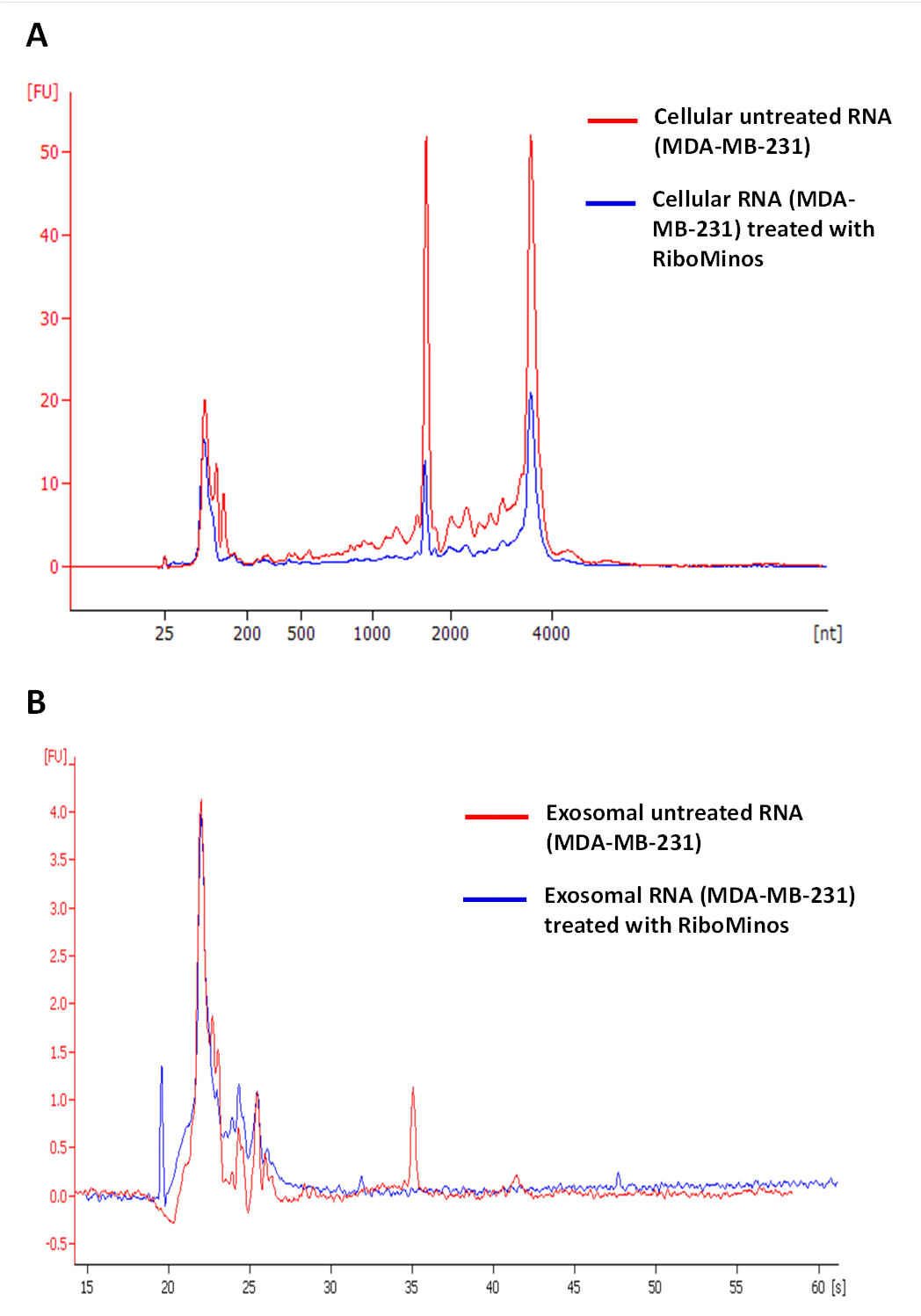

Supplement: Figure S4 — RNA was detected with PicoChip using Bioanalyzer. The depletion procedure has been performed according to the manufacturer’s protocol. Control samples (red) were treated exactly as experimental samples (blue) except they did not contain RiboMinus™ Probe. [file peerj-01-201-s004.png]

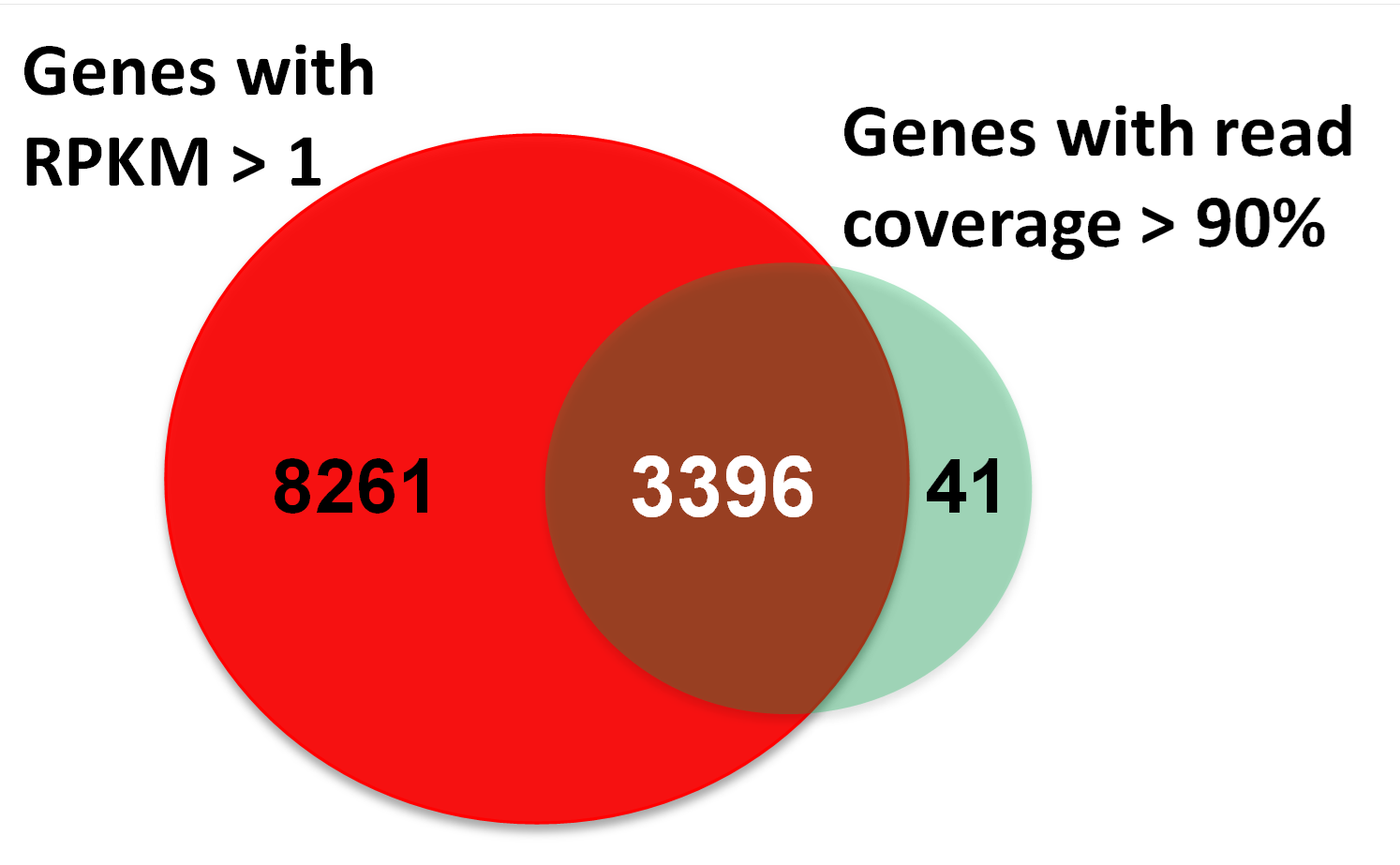

Supplement: Figure S5 — The detection criteria is that gene has more than 1 RPKM in at least one sample, while another approach is that gene has more than 90% coverage over protein-coding or non-coding sequence. [file peerj-01-201-s005.png]
